# Dr. Drake, on Digitalis

**Published:** 1800-04

**Authors:** Nathan Drake

**Affiliations:** Hadleigh, Suffolk


### *To the Editors of the Medical and Phyſical Journal.*

GENTLEMEN,

THE following correction of a miſquotation by Dr. Maclean, being introductory to a few facts with regard to the natural hiſtory of the Digitalis, I will thank you to give it a place in your valuable Journal. I am,

GENTLEMEN,

With great reſpect, your's, &c.

*Hadleigh, Suffolk, Feb.* 18, 1800. NATHAN DRAKE.

---

AS inaccuracy in quotation, eſpecially on controverted points, muſt neceſſarily diſcolour facts, and lead to concluſions totally unapprehended by the writer, it is incumbent upon every individual thus miſrepreſented, immediately to correct the error, whether ariſing from inadvertency or deſign.

Dr. Maclean has, in his laſt communication in your Journal, Vol. II. p. 151, when ſpeaking of a proper ſituation for the culture of the Digitalis, repreſented me as affirming that his houſe is damp. "If, the ſituation of my houſe," ſays he, "be certainly low and *damp*," as Dr. Drake has affirmed, it has hitherto eluded my own and my family's obſervation."

Now, if the Doctor will but advert to the paper in queſtion (for I readily ſuppoſe his miſtake to have ariſen from his truſting to memory) he will find no ſuch expreſſion or term as damp in the ſentence he refers to, nor the ſmalleſt alluſion to dampneſs, as applicable to his houſe. Thus is the ſentence written: "Dr. Maclean's houſe at Sudbury, is certainly ſituated low, not far from the river, and in the immediate neighbourhood of large chalk-beds." A ſituation which he has in common with the whole town of Sudbury, which is low, compared with the adjoining country, and built upon a chalky ſoil. This laſt circumſtance alone, had I not been previouſly acquainted with the ſcite of Sudbury, would have precluded, in my mind, any idea of dampneſs, which is, in general, irreconcileable with ſtrata of chalk.

Dr. Maclean has been led into this error by confounding two very oppoſite ſoils, though equally inimical to the growth of the Digitalis, and arbitrarily ſuppoſing I have applied them to his own ſituation; for in the paragraph preceding that he has miſquoted, it is affirmed, that Digitalis delights in an elevated, a light and gravelly ſoil; on a fat and denſe mould, in low and damp ſituations, and where ſtrata of chalk abound, it always

 degenerates, degenerates, becomes dwarfish, and of a pale green." Here a damp situation, a rich soil, and a chalky one, are distinct and opposed, though alike unfriendly to the plant in question; and the latter, frequently found in a low, but never in a damp scite, I have expressly declared to be that of the Doctor's immediate neighbourhood.

So far from the Digitalis being cultivated without difficulty, in any garden, which the Doctor assures those who may wish to possess it, is the case; he will, if he consult the writers upon the natural history of the plant, discover, that a *low* and *damp* soil, or a *chalky* soil, or a *fat* and *rich* soil, are highly detrimental to its growth; and that an *elevated* and *dry* soil, a *light* and *gravelly* soil, or a *barren* and *sandy* soil, are those in which it *alone* flourishes.

Dr. M. too, in insisting upon free exposure to the air, and freedom from adjoining plants, in cultivating the Digitalis, is, in another instance, deviating widely from the known habitudes of the wild plant. "These," observes he, "are often surrounded by weeds and other plants, which deprive them of a sufficient supply of light, air and nutriment from the earth, and thereby prevent them from attaining to full perfection."* Now, Botanical authors would inform him, that sheltered ground is the favourite situation of the Digitalis, which courts shade; is always found deep in hedge-rows; and even when growing on heaths or commons, the finest plants, as I well know through painful experience in gathering them, are found almost hidden by furze and bushes. That I may not be taxed, however, with general or unauthorized assertion, I shall conclude these remarks by quoting some of the first, both old and modern, writers on the natural history of the plant.

Nascitur in *montibus*, *umbrosis*, et *saxosis* locis. Fuschius De Digitali, cap. 342, p. 892, edit. 1542.

Fox-glove groweth in *barren sandy* grounds, and *under hedges*. Gerard, p. 647.

It is observable of this plant, that it grows *only on gravelly soils*; *rarely* or *never* on those where there are strata of *calcareous* earths. Lewis's Materia Medica, edition by Dr Aikin, 1791.

Digitalis. Place, *dry*, *gravelly* or *sandy*, soils. Withering, on Fox-glove, Introduction, p. 13.

---

* Medical and Physical Journal, Vol. II. p. 121.

